# Identification of NECTIN1 as a novel restriction factor for flavivirus infection

**DOI:** 10.1128/mbio.02708-24

**Published:** 2024-11-21

**Authors:** Shuhui Qi, Chao Sun, Jing Wang, Lijing Wo, Yongfeng Li, Chaonan Wang, Ying Zhang, Haiqiao Bian, Yongqi Guo, Ming Gao, Menghang Wang, Yandong Tang, Yuanmao Zhu, Fei Xue, Quanhai Pang, Zhigang Jiang, Xin Yin

**Affiliations:** 1 State Key Laboratory for Animal Disease Control and Prevention, Harbin Veterinary Research Institute, Chinese Academy of Agricultural Sciences, Harbin, China; 2 College of Veterinary Medicine, Shanxi Agricultural University, Taigu, Shanxi, China; 3 Molecular Biology, Teaching and Research Center, University of Liège, Gembloux, Belgium; Virginia Polytechnic Institute and State University, Blacksburg, Virginia, USA; Virginia Polytechnic Institute and State University, Blacksburg, Virginia, USA

**Keywords:** NECTIN1, BVDV, *Flaviviridae *family, CSFV, JEV, ZIKV, restriction factor

## Abstract

Nectin cell adhesion molecule 1 (NECTIN1) is a cell adhesion molecule that belongs to the immunoglobulin superfamily. It has been considered the most ubiquitous receptor for herpesviruses. However, in the context of flavivirus infection, its role was previously unknown. In this study, we described an arrayed siRNA screen mainly targeting Ig-like proteins that showed NECTIN1-restricted bovine viral diarrhea virus (BVDV) infection. We demonstrated that the depletion of NECTIN1 could significantly enhance the infection of both biotypes and multiple genotypes of BVDV, including BVDV-1a, -1b, -1c, -1p, -1m, -1v, and -2a. Notably, the IgV of NECTIN1 has emerged as the key domain restricting BVDV infection. Moreover, NECTIN1 inhibited BVDV attachment without exerting a significant influence on BVDV translation or transcription. Furthermore, we demonstrated that both NECTIN1 and CD46 could bind to BVDV E2, while the binding affinity of NECTIN1 for BVDV E2 was greater than that for CD46. We further identified that the BVDV E2 domain DD was a key domain of BVDV interacting with NECTIN1. In addition, we showed that NECTIN1 inhibited infections by classical swine fever virus (CSFV), Japanese encephalitis virus (JEV), and Zika virus (ZIKV), which belong to the *Flaviviridae* family, but had limited effects on bluetongue virus (BTV), vesicular stomatitis virus (VSV), Akabane virus (AKAV), and Sindbis virus (SINV). Overall, our study has important implications for understanding the entry of BVDV and revealed a novel role for NECTIN1 as a restriction factor that inhibits flavivirus infection.

**IMPORTANCE:**

NECTIN1, also known as CD111 or PVRL1, has been recognized as the primary receptor for several alpha herpesviruses, including herpes simplex virus (HSV), pseudorabies virus (PRV), and bovine herpesvirus 1 (BHV-1). However, our study revealed a novel role for NECTIN1 in the virus life cycle by influencing BVDV infection. Contrary to its role as a receptor for alpha herpesviruses, NECTIN1 acts as a restriction factor for BVDV by inhibiting viral attachment via competition with CD46 for binding to the domain DD of BVDV E2. We further revealed that the replication of members of the *Flaviviridae* family was inhibited by NECTIN1, while the replication of other RNA viruses did not significantly differ. Our results demonstrate that NECTIN1 is a novel factor restricting *Flaviviridae* family virus replication and highlight the complexity of virus-host interactions and the multifaceted nature of host factors involved in viral infection.

## INTRODUCTION

The NECTIN/NECL family comprises a group of cell adhesion molecules that play essential roles in mediating cell-cell adhesion and communication (1). Besides NECTIN1, other members of this family include NECTIN2, NECTIN3, NECTIN4, NECL1, NECL2, NECL3, NECL4, and NECL5 (1, 2). These transmembrane proteins belonging to the immunoglobulin (Ig) superfamily are involved in various cellular processes, such as the formation of adherens junctions, signal transduction, and tissue organization (3). Each member of the NECTIN family exhibits distinct expression patterns and functions, contributing to the diversity of cellular adhesion mechanisms (1, 2). NECTIN1, also known as CD111 (4), stands out as a crucial receptor for alpha herpesviruses such as herpes simplex virus type 1 (HSV-1) (5, 6) and herpes simplex virus type 2 (HSV-2) (6). Its interaction with viral glycoprotein D facilitates viral infection and replication (7). In addition, NECTIN1 is involved in the regulation of immune responses (8), neuronal development (9), and cancer progression (10, 11), highlighting its multifaceted functions in both health and disease contexts.

*Flaviviridae* family is mainly classified into four genera: *Flavivirus*, *Pestivirus*, *Hepacivirus*, and *Pegivirus,* the members of which share similar characteristics and genetic relationships. Bovine viral diarrhea virus (BVDV) is a typical member of the *Pestivirus* genus which seriously threatens the development of the breeding industry (12, 13). Furthermore, BVDV can infect many species of animals of the order *Artiodactyla* including cattle, sheep, pigs, camels, and goats, with multi-tissue tropism (14–16). Like other members of the *Flaviviridae* family, the BVDV genome is nearly 12.5 kb in length and encodes 12 proteins, four structural proteins C, E^rns^, E1, and E2, which mainly participate in viral particle assembly, and eight non-structural proteins N^pro^, P7, NS2, NS3, NS4A, NS4B, NS5A, and NS5B, which participate in viral replication (17). Notably, the envelope protein E2 can form the homodimers E2-E2 and heterodimers E1-E2, which mediate interactions with host receptors and play important roles in BVDV entry (18). However, the detailed functional roles of these viral proteins in the BVDV lifecycle still need to be discovered.

Virus entry, involving attachment to and penetration into the host cells, marks the initial step in the virus life cycle. Thus far, four host proteins have been identified as cellular factors for BVDV entry, namely complement regulatory protein 46 (CD46) (19), heparan sulfate (HS) (20), the low-density lipoprotein (LDL) receptor (21), and a disintegrin and metalloproteinase 17 (ADAM17) (22). Among them, CD46 is the main cellular receptor mediating BVDV entry by interacting with E2. Notably, CD46 belongs to the immunoglobulin superfamily and serves as a regulator of the complement system by cleaving complement components such as C3b and C4b. Moreover, several host receptors for flaviviruses, including AXL (23), CD209 (24), TYRO3 (25), and TIM-1 (26) also belong to the immunoglobulin superfamily and share a similar structure with CD46. Therefore, it seems that proteins with an Ig-like structure play a broad role in flavivirus infections. Nevertheless, further studies are urgently needed to uncover the intricacies of these interactions.

In this study, we found that Ig-like protein NECTIN1, known as the primary receptor for alpha herpesvirus, instead exhibited antiviral activities against BVDV infection. We further demonstrated that NECTIN1 mainly blocked BVDV entry at the early stage of the lifecycle and that the IgV domain was vital for its anti-BVDV activity. NECTIN1 can bind to BVDV E2 by competing with CD46, thereby limiting the attachment of BVDV. Depletion of NECTIN1 in primary bovine cells resulted in a significant increase in BVDV replication. Importantly, NECTIN1 also exhibited antiviral activity against classical swine fever virus (CSFV), Japanese encephalitis virus (JEV), and Zika virus (ZIKV) infections. In conclusion, in addition to serving as a receptor, NECTIN1 also functions as a restriction factor in BVDV infection. These findings suggest intriguing research directions for further elucidating the role of NECTIN1 in regulating host cell infection by viruses of the *Flaviviridae* family.

## MATERIALS AND METHODS

### Cells and cell culture

HEK-293T cells, Madin-Darby bovine kidney (MDBK) cells, Chinese hamster ovary (CHO) cells, PK-15 cells, bovine turbinate cells, bovine tracheal cells, and Huh7.5.1 cells were preserved in the laboratory and cultured in custom medium that Dulbecco’s modified Eagle’s medium (DMEM) supplemented with 10% heat-inactivated fetal bovine serum (FBS), 6 mM L-glutamine, penicillin (100 U/mL), and streptavidin (100 µg/mL). NECTIN1-FLAG-overexpressing MDBK cells were generated by infection with packaged lentivirus, screened with puromycin, and cultured in a custom medium with puromycin (2 µg/mL). NECTIN1 knockout cell lines were obtained by infection with packaged lentivirus, and single cells were sorted after screening with blasticidin and cultured in a custom medium with blasticidin (6 µg/mL). Cells expressing the NECTIN1 sgRNA mutant and truncated fragments (Δsignal/ΔIgV/ΔIgC1/ΔIgC2/ΔTM/ΔPDZ) were generated by infection with packaged lentivirus and as described above screened after doxycycline induction.

### Plasmids, siRNAs, and transfection

pCAGGS-bovine CD46-myc was a gift from Dr. Su Li at Harbin Veterinary Research Institute. pLVX-IRES-puro-NECTIN1-2×HA-3×FLAG was synthesized and constructed by RuiBiotech (Beijing, China). The bovine NECTIN1 cDNA was PCR amplified from MDBK cells, then cloned and inserted into pCAGGS-2×HA and pCAGGS-3×FLAG, separately. The NECTIN1 sgRNA-mutant was amplified, cloned, and inserted into pLVX-TetOne-Puro-3×FLAG. Based on the NECTIN1 sgRNA mutant, the NECTIN1 Δsignal/ΔIgV/ΔIgC1/ΔIgC2/ΔTM/ΔPDZ strains were amplified, cloned, and inserted into pLVX-TetOne-Puro-3×FLAG. The three sgRNAs for NECTIN1 were separately cloned and inserted into lentiCas9-blast. All plasmids were confirmed by DNA sequencing by the RuiBiotech (Beijing, China). All siRNAs and control scrambled siRNAs were purchased from GenePharma (Shanghai, China). All transfections were carried out using PEI MAX-Transfection Grade Linear Polyethylenimine Hydrochloride (MW 40,000) (Polysciences, Warrington, PA, USA) for DNA plasmids or Lipofectamine RNAiMAX (Invitrogen, Carlsbad, CA, USA) for siRNA according to the manufacturer’s instructions.

### Viruses

BVDV-1b, BVDV-1c, BVDV-1v, BVDV-1m, and BVDV-1p were stored in our laboratory, and VSV-GFP, HSV-1-GFP, BTV-GFP, and AKAV-wasabi were generated in our laboratory. The infection clone BVDV-1a NADL-mCherry was provided by Dr. Diego E. Alvarez. BVDV-2a was a gift from Dr. Mingchun Gao (27), JEV-GFP and SINV-GFP were kindly provided by Dr. Yanhua Li at Yangzhou University and Dr. Margaret MacDonald at Rockefeller University, respectively. The CSFV-sm strain was a gift from Dr. Huaji Qiu at Harbin Veterinary Research Institute, and ZIKV was a gift from Dr. Wanbo Tai at Shenzhen Bay Laboratory.

### Reagents and antibodies

Doxycycline and puromycin dihydrochloride were purchased from Beyotime (Shanghai, China). Blasticidin was purchased from Solarbio (Beijing, China). Anti-FLAG M2 magnetic beads affinity isolated antibody was purchased from Sigma-Aldrich (Shanghai, China). The following antibodies were used: primary antibodies BVDV type 1 and 2 (BVDV-1&2) MAb E2 gp53 IgG2b isotype (1:1,000, 348, VMRD), bovine viral diarrhea virus (BVDV) antiserum (1:1,600, PAB-BVD, VMRD), anti-dsRNA MAb (IgG2a) (1:1,000, 10010200, SCICONS), DYKDDDDK tag monoclonal antibody (1:1,000 for IFA, 1:10,000 for WB, 66008-4-Ig, Proteintech), HA tag polyclonal antibody (1:100 for IFA, 1:1,000 for WB, 51064-2-AP, Proteintech), MYC tag polyclonal antibody (1:200 for IFA, 1:1,000 for WB, 16286-1-AP, Proteintech), the anti-BVDV NS3 monoclonal antibody saved by the laboratory, and beta-actin monoclonal antibody from mouse (1:10,000, 66009-1-Ig, Proteintech). Secondary antibodies included that goat anti-mouse/rabbit IgG (H+L) highly cross-adsorbed secondary antibody, Alexa Fluor 488 (1:1,000, A-11029/A-11034, Invitrogen), goat anti-mouse/rabbit IgG (H+L) highly cross-adsorbed secondary antibody, Alexa Fluor 568 (1:1,000, A-11031/A-11011, Invitrogen), donkey anti-goat IgG (H+L) highly cross-adsorbed secondary antibody, Alexa Fluor Plus 488 (1:1,000, A32814, Invitrogen), IRDye 800CW goat anti-mouse IgG secondary antibody (1:10,000, 926-32210, LI-COR Biosciences), IRDye 800CW donkey anti-rabbit IgG secondary antibody (1:10,000, 926-32213, LI-COR Biosciences), and IRDye 680RD goat anti-mouse/anti-rabbit IgG secondary antibody (1:10,000, 926-68070/926-68071, LI-COR Biosciences).

### Cell viability assay

Three NECTIN1 knockout cell lines and MDBK cells were seeded into 96-well plates at 1 × 10^3^/well for different durations (24, 36, 48, 60, 72, and 96 h). Afterward, 10 µL of CCK-8 solution was added to each well at each time point, and the cells were incubated for 1 h in a cell culture incubator. Then, the absorbance at 450 nm was measured using a microplate reader, and the absorbance was analyzed with GraphPad.

### Immunofluorescence assay

The cells were washed with PBS and fixed with 4% paraformaldehyde and permeabilized by 0.2% Triton X-100. Then, the cells were incubated with primary antibodies after blocking by 1% BSA at 4°C overnight. The next day, the cells were washed with PBS and then incubated with secondary antibodies for 45 min at 37°C. The nuclei were stained with DAPI for 15 min at room temperature. Images were acquired by inverted fluorescence microscopy with an EVOS M5000 (scale bars: 300 µm) and confocal laser scanning microscopy with Airyscan LSM800 (scale bars: 5 µm). The number of dsRNA granules in the cells was automatically calculated with ImageJ software.

### Immunoprecipitation analysis of the interaction between BVDV E2, CD46, and NECTIN1

To demonstrate the relationships among BVDV E2, CD46, and NECTIN1, the above three plasmids were co-transfected into HEK293T cells in pairs into three groups, and the other group was co-transfected with the above three plasmids into HEK293T cells. The NECTIN1 plasmid was transfected in a dose-dependent manner (such as 0, 0.5, 1, 1.5, 2, and 2.5 µg). After transfection for 48 h, the cells were washed with 1× PBS three times and then lysed cells with lysis buffer containing 150 mM NaCl, 50 mM Tris-HCl (pH 8.0), 0.8% NP-40, and PMSF (100 µM) for 30 min at 4℃. Then, the cells were centrifuged, and the cell lysates were collected and incubated on a shaker with 10 µL of anti-FLAG magnetic beads (Sigma) at 4°C overnight. The next day, the beads were boiled with 1× loading buffer for 10 min at 100°C after being washed five times with lysis buffer and then analyzed by western blot.

### Western blot assay

Cells were harvested using lysis buffer. Equivalent protein aliquots were subjected to SDS-PAGE and transferred to NC membranes. The membranes were then blocked with 5% fat-free milk for 1 h at room temperature and probed with primary antibodies, followed by the corresponding near-infrared fluorescent dye-labeled secondary antibodies such as goat anti-mouse or goat anti-rabbit secondary antibodies (LI-COR Biosciences, Lincoln, NE, USA).

### BVDV-NADL replicon and BVDV-NADL replicon-NS5B-GAA transfection and NanoLuc detection

The pACNR-BVDV-NADL-mCherry was used as a backbone vector to construct the BVDV replicon. As described in previous studies (28, 29), we replaced BVDV structural proteins (capsid, E^rns^, E1, and E2) and nonstructural proteins (P7 and NS2) with the Nanoluc reporter gene to create the BVDV replicon. Based on this replicon construct, we mutated the key active sites in NS5B-GDD, resulting in the BVDV-NADL-replicon-NS5B-GAA mutant. All these BVDV replicon constructs were digested with *Sbf* I and purified by adding 1/10 volume of 3 M NaOAc and 3 volumes of cold ethanol. Then the DNA products were chilled at −20°C overnight, followed by centrifugation for 30 min at 15,000 rpm to pellet the DNA. The supernatant was subsequently removed, and the remaining pellets were resuspended in ddH_2_O. Next, the purified linearized plasmids were transcribed *in vitro* using mMESSAGE mMACHINE T7 Transcription Kit. The transcription reaction was terminated by the addition of TURBO DNase for 15 min at 37°C. The transcribed RNA was then extracted with phenol-chloroform-isoamyl alcohol (PCA) and precipitated with isopropyl alcohol. After drying, the RNA was redissolved in RNase-free water, and its concentration of RNA was determined by measuring the optical density using a Nanodrop spectrophotometer. For electroporation, 8 µg of the transcribed RNA was electroporated into 1.6 × 10^6^ cells (conditions: 180 V, 950 µF, 2 mm gap cuvette). Immediately after electroporation, the cells were resuspended in complete DMEM and seeded as required into 24-well plates (2 × 10^5^ cells per well). At indicated time points post-electroporation, cell lysates were harvested and analyzed through the luciferase activity (30).

### The generation of CRISPR-Cas9-mediated knockout cell lines

We constructed NECTIN1 knockout cell lines by lentivirus infection, the single-guide RNA (sgRNA) sequence 5′-CATGTACGGTTTCATCGGCA-3′ (selected from the Zhang Laboratory website) and cloned them into the pLentiCRISPR v2 plasmid and then sorted single cells after screened with blasticidin. Finally, the NECTIN1 knockout cells were confirmed by DNA sequencing.

### Binding assay

NECTIN1 KO1 and MDBK WT cells were seeded into 24-well cell plates at 2 × 10^5^/well. After 16 h post-seeding, the cells were incubated with BVDV at MOI = 0.05, 0.5, and 5 separately for 1 h on ice. The inoculated cells were then washed five times with cold PBS, and subjected to RNA extraction for qPCR. The data were analyzed with the 2^−ΔΔCT^ method (31).

### Surface plasmon resonance assays

The gene encoding bovine NECTIN1 was cloned into the plasmid pTT5 vector with 6×his tag. The protein was then expressed in CHO cells and purified through a nickel column. The gene encoding BVDV E2 was cloned into the plasmid pCAGGS vector with 6×his tag. BVDV E2 protein was expressed using 293F cells transfected with the expression construct and purified through a nickel column as reported previously (32). The bovine CD46 protein was purchased from MedChemExpress (MCE).

The binding kinetics of BVDV E2 with NECTIN1 or CD46 were accessed using surface plasmon resonance (SPR) analysis on a Biacore 8K system (GE Healthcare) with Biacore Series S Sensor Chip CM5 (Cytiva Life Sciences) at 25°C in single-cycle mode. All proteins used in the kinetic analysis were exchanged into the PBS buffer. NECTIN1 and CD46 proteins were captured using the Biacore Series S Sensor Chip CM5, and serial dilutions of BVDV E2 were injected onto the chip surface to access binding. After each cycle, the sensor chips were regenerated using glycine (pH 2.5). The *K*_*D*_ values were calculated using Biacore 8K evaluation software (GE Healthcare). Figures were generated using GraphPad Prism 9.4.

### Plasma membrane separation assay

The HEK 293T were seed in six-well cell plates and transfected with NECTIN1-2HA and CD46-MYC at 5 µg/well, 48 h post-transfection, the cells were collected for separation of plasma membrane protein and non-plasma membrane protein with Minute Plasma Membrane/Protein Isolation and Cell Fractionation Kit (50 Preps) (Invent Biotechnologies) (33, 34) and then detected by western blot.

### The protein interactions predicted by AlphaFold 3

The crystal structural model of NECTIN1-bovine protein and BVDV E2 protein was constructed by AlphaFold 3. The complex structures of the NECTIN1-bovine with the BVDV E2 were determined by AlphaFold 3 and analyzed by Schrödinger (35).

### Statistical analysis and image generation

Statistical significance was calculated using a *t*-test (unpaired/paired) or one-way or two-way analysis of variance (ANOVA) followed by Dunnett’s post hoc test for multiple comparisons. Figure layouts were generated using Adobe Photoshop CS5. Schematic illustrations were generated using BioRender (https://www.biorender.com/library).

## RESULTS

### NECTIN1 inhibits BVDV infection according to siRNA pool screening

**Fig 1:**
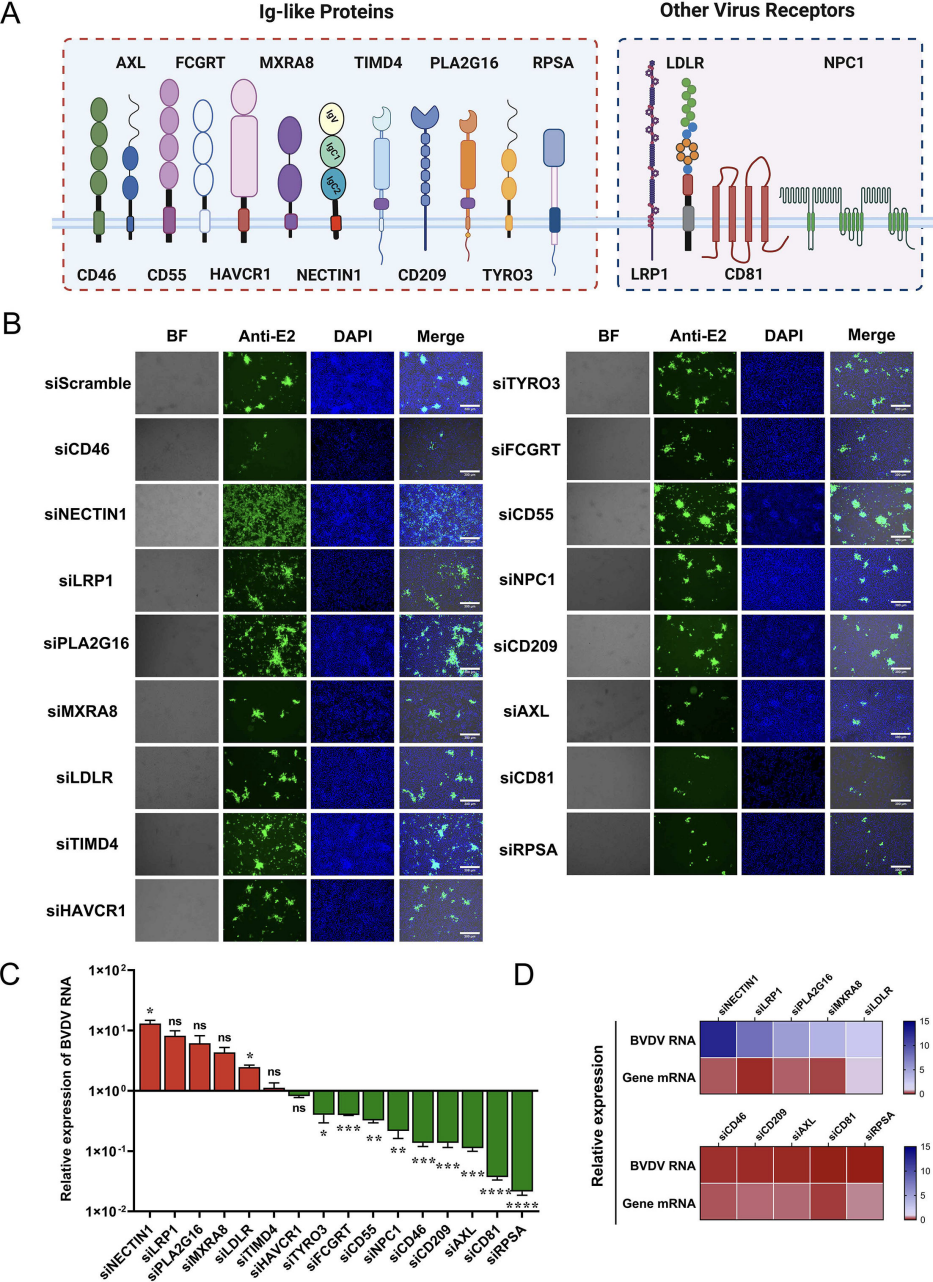
siRNA pools targeting membrane protein genes have been identified as important host factors for BVDV infection. (**A**) Schematic representation of the 16 candidate proteins for BVDV replication which is targeted by siRNA pools. (**B**) IFA identification of BVDV infection levels after knockdown with the siRNA pool. The cells were infected with BVDV at an MOI of 1 and harvested at 48 h postinfection (hpi.). Scale bar, 300 µm. (**C**) Quantitative reverse-transcriptase PCR (qPCR) analysis of total BVDV RNA levels following knockdown with the siRNA pool. siScramble was used as the control. The cells were infected with BVDV at an MOI of 1 and harvested at 48 hpi. (**D**) The top five genes identified in the screening either inhibit or promote BVDV infection, which normalized against RNA levels in cells transfected with scramble siRNA.

To identify novel membrane proteins that regulate BVDV infection, we selected 16 candidates (CD46 was used as a positive control) for RNAi screening in MDBK cells, a cell line permissive to BVDV infection. 12 of the 16 candidates were Ig-like proteins (such as CD55, NECTIN1, and TIMD4) that share similar structures with CD46, and the remaining candidates were cellular receptors for viruses belonging to the *Flaviviridae* family or other viruses (Fig. 1A) (36–39). Following siRNA transfection, the MDBK cells were infected with BVDV at an MOI of 1. Two days later, the infected cells were either stained with an anti-BVDV monoclonal antibody or subjected to viral RNA quantification to determine the BVDV infection rate. We found that the knockdown of multiple genes, including RPSA, CD81, AXL, and CD209, significantly reduced cytopathic (CP) BVDV strain infection, suggesting the potential role of these genes as proviral or attachment factors (Fig. 1B and C). Interestingly, depleting NECTIN1, LRP1, PLA2G16, MXRA8, and LDLR instead led to an increase in the BVDV E2 staining signal and genomic load in MDBK cells (Fig. 1B through D), demonstrating that these factors have antiviral functions in BVDV infection. Notably, among these antiviral candidates, we observed that siRNA-mediated depletion of NECTIN1 resulted in a substantial 13-fold increase in the BVDV genomic load compared with that of siScramble (Fig. 1C). Given that NECTIN1 is a well-documented receptor for alpha herpesviruses, including herpes simplex virus type 1 (HSV-1), pseudorabies virus (PRV), and bovine herpesvirus-1 (BHV-1) (40, 41), we focused our subsequent investigation on NECTIN1.

### NECTIN1 is a pan-BVDV restriction factor

**Fig 2:**
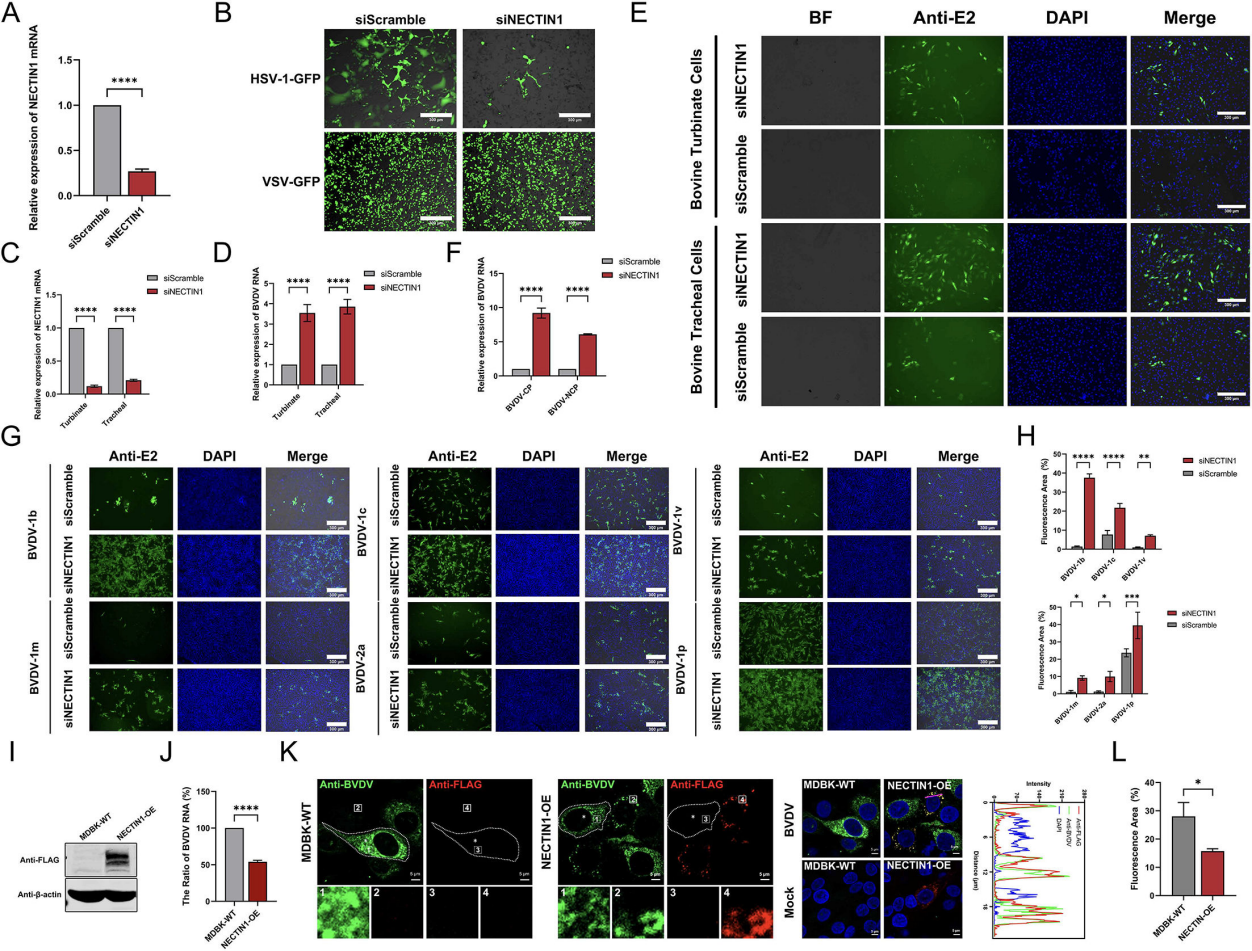
NECTIN1 acts as a limiting factor for BVDV replication. (**A**) qPCR quantification of NECTIN1 mRNA levels after knockdown. siScramble was used as a control. (**B**) Infection with HSV-1-GFP and VSV-GFP after NECTIN1 knockdown. The cells were infected with BVDV at an MOI of 0.1 and harvested at 12 hpi. Scale bar, 300 µm. (**C**) qPCR quantification of NECTIN1 mRNA levels in different bovine cell lines following NECTIN1 knockdown. siScramble was used as a control. (**D**) qPCR quantification of the BVDV RNA levels in different bovine cell lines following NECTIN1 knockdown. siScramble was used as a control. The cells were infected at an MOI of 0.1 and harvested at 24 hpi. (**E**) IFA analysis of BVDV replication in different bovine cell lines after NECTIN1 knockdown. The cells were infected at an MOI of 0.1 and harvested at 24 hpi. Scale bar, 300 µm. (**F**) qPCR quantification of the RNA levels of the two biotypes of BVDV following NECTIN1 knockdown. siScramble was used as a control. The cells were infected at an MOI of 1 and harvested at 48 hpi. (**G**) IFA analysis of various genotypes of BVDV replication following NECTIN1 knockdown. Cells were infected BVDV-1p at MOI of 1, or BVDV-1c and BVDV 2a at MOI of 0.5, or BVDV 1m, BVDV 1b, and BVDV-1v at MOI of 0.1. Scale bar, 300 µm. (**H**) The calculation of the fluorescence area (%) where the different genotypes of BVDV cause cellular cytopathy after NECTIN1 knockdown. (**I**) Western blot analysis confirming NECTIN1 overexpression. (**J**) The ratio of BVDV RNA expression after NECTIN1 overexpression. MDBK-WT was used as a control. The cells were infected at an MOI of 1 and harvested at 48 hpi. (**K**) Confocal identification of BVDV infection after NECTIN1 overexpression. The cells were infected at an MOI of 1 and harvested at 48 hpi. The fluorescence signal in the pink line area was analyzed by the intensity peak diagram. Scale bar, 5 µm. (**L**) The calculation of the fluorescence area (%) where the different genotypes of BVDV cause cellular cytopathy after NECTIN1 overexpression.

To further investigate the role of NECTIN1 in the BVDV lifecycle, we first confirmed the knockdown efficiency of NECTIN1 by qPCR and infected the knockdown cells with HSV-1-GFP to prove its loss of function. As shown in Fig. 2A, the amount of NECTIN1 was reduced by nearly 70% upon NECTIN1 siRNA transfection compared to that in the control group. Consistently, HSV-1-GFP infection was also significantly mitigated by depletion of NECTIN1, whereas no obvious impact on infection with an irrelevant virus (VSV-GFP) was observed under the same treatment (Fig. 2B). To avoid these results due to the specific nature of MDBK cells, we further infected the turbinate cells and tracheal cells with the BVDV-CP strain after NECTIN1 interference. As shown in Fig. 2C and E, compared with that in siScramble cells, BVDV replication was significantly increased in cells with NECTIN1 depletion. These results confirmed that enhanced BVDV infection resulted from the loss of NECTIN1 in siRNA-transfected cells. Since BVDV is classified into two biotypes (CP and NCP), we also determined the antiviral activities of NECTIN1 against these two BVDV biotypes. Viral RNA quantification revealed that NECTIN1 significantly inhibited the proliferation of both the BVDV-CP and BVDV-NCP strains (Fig. 2F). Furthermore, we likewise verified whether NECTIN1 broadly inhibits different genotypes of BVDV (BVDV-1 and BVDV-2). We collected BVDV strains of different genotypes from our laboratory and infected cells following NECTIN1 knockdown. The results from the immunofluorescence assay (IFA) and quantification of the fluorescent area (%) of three images revealed that the replication of all these test genotypes was significantly elevated (Fig. 2G and H). Overall, we concluded that NECTIN1 is a pan-BVDV restriction factor.

To further establish the role of NECTIN1 as a limiting factor for BVDV infection, we constructed an MDBK cell line stably expressing NECTIN1. The protein expression was determined by western blot (Fig. 2I). Subsequently, we compared the BVDV genome levels between NECTIN1-overexpressing MDBK cells and parental MDBK cells, as shown in Fig. 2J. The results indicated a twofold decrease in the abundance of the BVDV genome in the NECTIN1-overexpressing cells compared to the parental cells. Moreover, we performed confocal imaging of MDBK cells overexpression with NECTIN1 followed by BVDV infection and found that the BVDV staining signal was much weaker in NECTIN1-overexpressing cells (Fig. 2K and L). Interestingly, BVDV was restricted to a specific area containing NECTIN1 (Fig. 2K). These results further demonstrated that NECTIN1 restricts BVDV infection.

### Members of the NECTIN/NECL family diversely regulate BVDV infection

**Fig 3:**
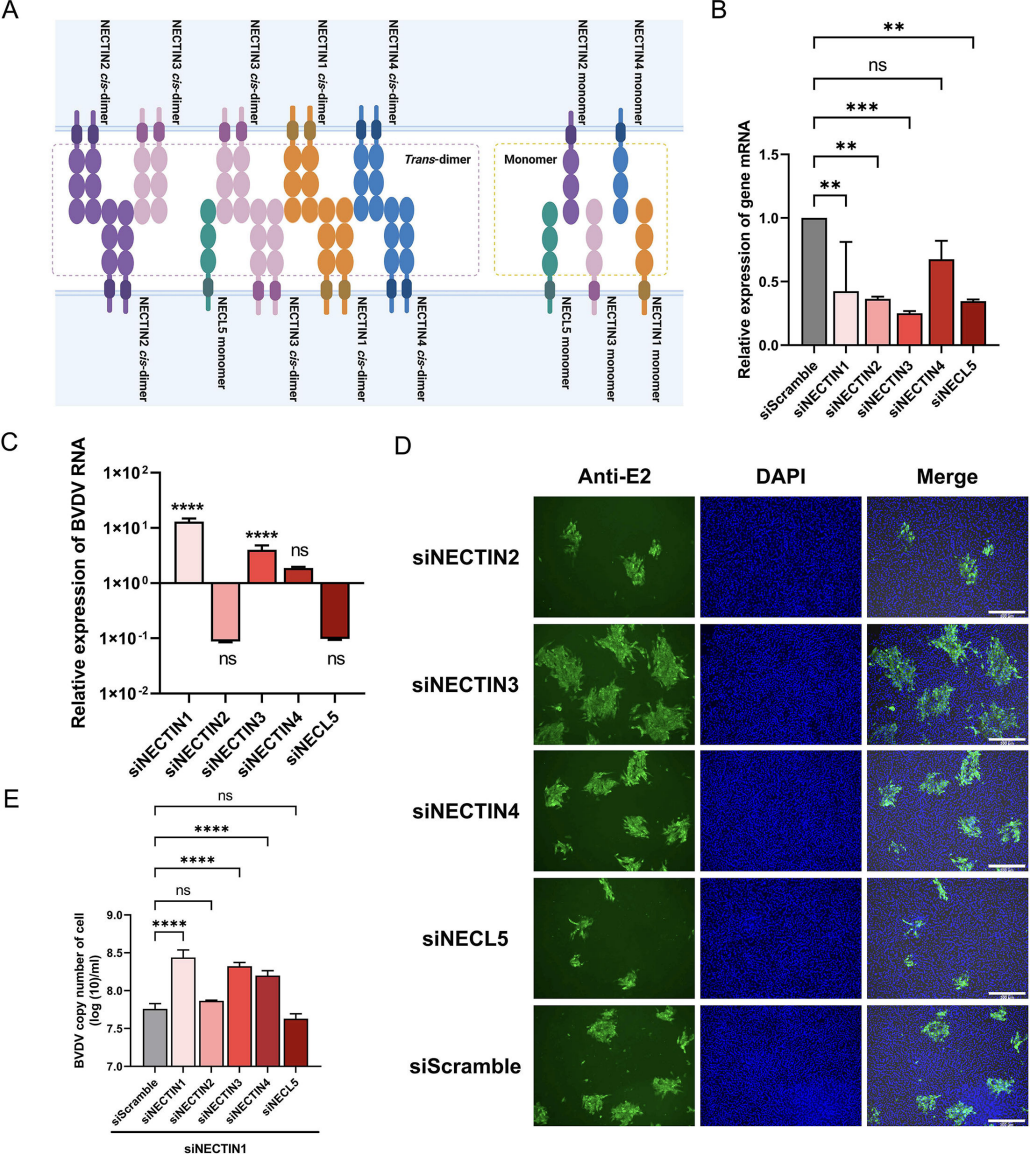
NECTIN/NECL family proteins exhibit varied effects on BVDV replication. (**A**) Schematic illustration of NECTIN/NECL family protein interactions. (**B**) qPCR quantification of the mRNA levels of NECTIN/NECL family members after knockdown. (**C**) qPCR quantification of BVDV RNA levels following the knockdown of NECTIN/NECL family members. As the baseline, the value of siScramble was used as a control. The cells were infected at an MOI of 1 and harvested at 48 hpi. (**D**) IFA identification of BVDV infection after the knockdown of NECTIN/NECL family members. The cells were infected at an MOI of 1 and harvested at 48 hpi. Scale bar, 300 µm. (**E**) qPCR quantification of BVDV RNA levels after double knockdown of NECTIN1 and NECTIN/NECL family members. siScramble was used as a control. The cells were infected at an MOI of 1 and harvested at 48 hpi.

NECTIN1 is a member of the immunoglobulin-like cell adhesion molecule (CAM) family and belongs to the NECTIN family. All the members of the NECTIN/NECL family possess the same protein structure, which consists of a cytoplasmic tail, a single transmembrane region, and an extracellular region with three Ig-like domains. Notably, NECTIN/NECL family proteins can form homo- or heterodimers (Fig. 3A). Therefore, the roles of members of the NECTIN/NECL family in BVDV infection warrant comprehensive investigation. To this end, we selected NECTIN2, NECTIN3, NECTIN4, and NECL5 (NECTIN-like molecule 5) to assess their function in BVDV replication via siRNA-mediated knockdown (Fig. 3B). We found that knockdown of NECTIN3 and NECTIN4 moderately enhanced BVDV infection, as measured by virus RNA quantification and BVDV E2 staining, whereas there was non-significant difference in BVDV RNA levels after depletion of NECTIN2 and NECL5 (Fig. 3C and D). NECTIN1 can form heterodimers by binding with NECTIN3 or NECTIN4. We further determined whether other NECTIN proteins synergize with NECTIN1 to inhibit BVDV infection. As shown in Fig. 3E, depletion of NECTIN3 and NECTIN4 rather than NECTIN2 or NECL5 still enhanced BVDV infection in siNECTIN1 cells, suggesting that the function of NECTIN3/NECTIN4 in BVDV replication was not influenced by NECTIN1 and the formation of heterodimers between NECTIN1 and NECTIN3/NECTIN4 was not required for BVDV replication.

### The IgV and TM domains of NECTIN1 are vital for its antiviral activity against BVDV infection

**Fig 4:**
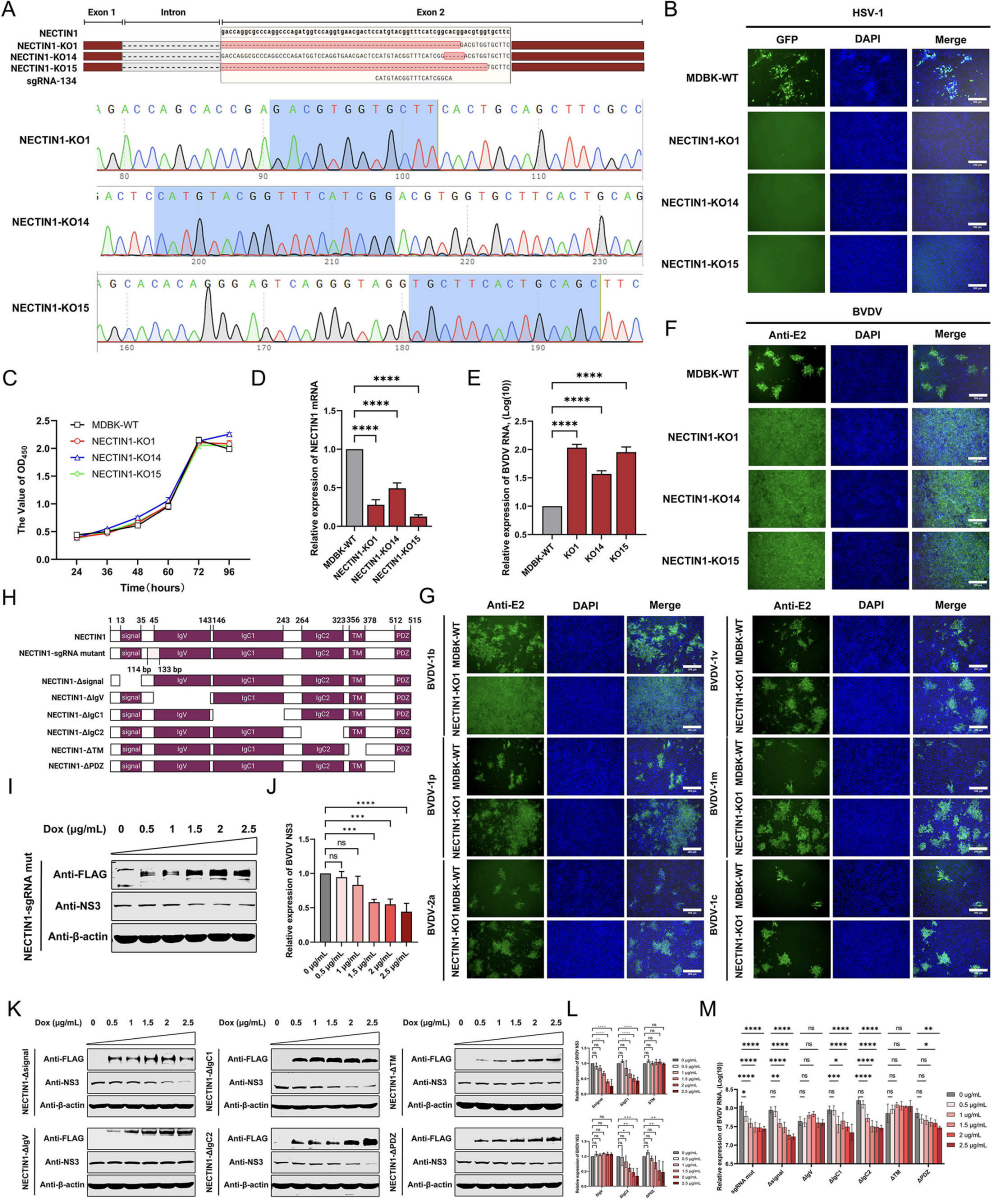
BVDV replication is increased in NECTIN1 knockout cell lines. (**A**) A schematic diagram of the NECTIN1 knockout cell line sequence and a diagram of the sequencing peaks of the NECTIN1 knockout cells. (**B**) Infection of NECTIN1 knockout cell lines with HSV-1-GFP. The cells were infected at an MOI of 0.1 and harvested at 12 hpi. Scale bar, 300 µm. (**C**) Growth curves of NECTIN1 knockout cell clones and MDBK-WT cells. (**D**) qPCR quantification of NECTIN1 mRNA levels after knockout. MDBK-WT was used as a control. (**E**) qPCR quantification of BVDV RNA levels in NECTIN1 knockout cell clones (NECTIN1-KO1, NECTIN1-KO14, and NECTIN1-KO15). MDBK-WT was used as a control. The cells were infected at an MOI of 1 and harvested at 48 hpi. (**F**) IFA identification of BVDV infection in MDBK-WT and NECTIN1 knockout cell clones. MDBK-WT was used as a control. The cells were infected at an MOI of 1 and harvested at 48 hpi. Scale bar, 300 µm. (**G**) Identification of BVDV various genotypes infection via IFA in the NECTIN1-KO1 cell clone. MDBK-WT was used as a control. Cells were infected with BVDV-1b at MOI of 1, or BVDV-1v, BVDV 2a, BVDV 1m, BVDV 1c, and BVDV-1p at MOI of 0.5. Scale bar, 300 µm. (**H**) Schematic diagram of NECTIN1 sgRNA mutants and six truncated forms of NECTIN1. (**I and J**) Western blot analysis of the BVDV replication level and gray value analysis after NECTIN1 sgRNA mut complementation. Dox at a concentration of 0 µg/mL was used as a control. (**K and L**) Western blot analysis of the BVDV replication level and gray value analysis after the complementation of six truncated NECTIN1 strains. Dox at a concentration of 0 µg/mL was used as a control. (**M**) qPCR quantification of BVDV RNA levels in cells overexpressed with NECTIN1 sgRNA mutants and six truncated NECTIN1 constructs. Dox at a concentration of 0 µg/mL was used as a control.

To further identify the domains within NECTIN1 required for its anti-BVDV functions, we constructed NECTIN1 knockout cell lines through CRISPR/Cas9 gene editing methods. Finally, three NECTIN1 knockout cell clones, namely, NECTIN1-KO1, NECTIN1-KO14, and NECTIN1-KO15, were obtained upon validation via sequence analysis (Fig. 4A) and loss-of-function detection (Fig. 4B). The cell viability results showed that the loss of NECTIN1 had no impact on cell growth (Fig. 4C). The mRNA level in the NECTIN1 knockout cell clones was significantly lower than that in the WT cells (Fig. 4D). Consistent with the results obtained in the context of siRNA-mediated NECTIN1 depletion, BVDV infection was dramatically enhanced in the cell clones in which NECTIN1 was knocked out. The level of BVDV RNA in these infected cells was at least 36-fold greater than that in MDBK-WT cells (Fig. 4E). The IFA results showed that the level of BVDV replication in the NECTIN1 knockout cells was significantly greater than that in the MDBK-WT cells (Fig. 4F and G). Doxycycline-induced expression of the NECTIN1 mutant bearing a mutation at the sgRNA recognition site in the knockout cell clone led to reduced BVDV infection in a dose-dependent manner (Fig. 4H through J). As mentioned above, NECTIN1 is composed of a cytoplasmic tail, a single transmembrane region, and an extracellular region with three Ig-like domains (42). To identify the domains required for anti-BVDV infection, we generated cell lines expressing truncated NECTIN1 under the control of doxycycline using the NECTIN1-KO1 cell clone as the parental line and then assessed the BVDV infection in each cell line. As shown in Fig. 4K and L, the replication of BVDV decreased in cells expressing the mutants NECTIN1-Δsignal, NECTIN1-ΔIgC1, NECTIN1-ΔIgC2, and NECTIN1-ΔPDZ, whereas the expression of NECTIN1-ΔIgV and NECTIN1-ΔTM had no impact on BVDV replication. The quantification of viral RNA using BVDV-specific qPCR revealed a pattern consistent with that observed in the western blot analysis (Fig. 4M). Collectively, these results demonstrated that both the IgV and TM domains within NECTIN1 were required for its antiviral activity against BVDV.

### NECTIN1 interferes with BVDV entry during BVDV infection

**Fig 5:**
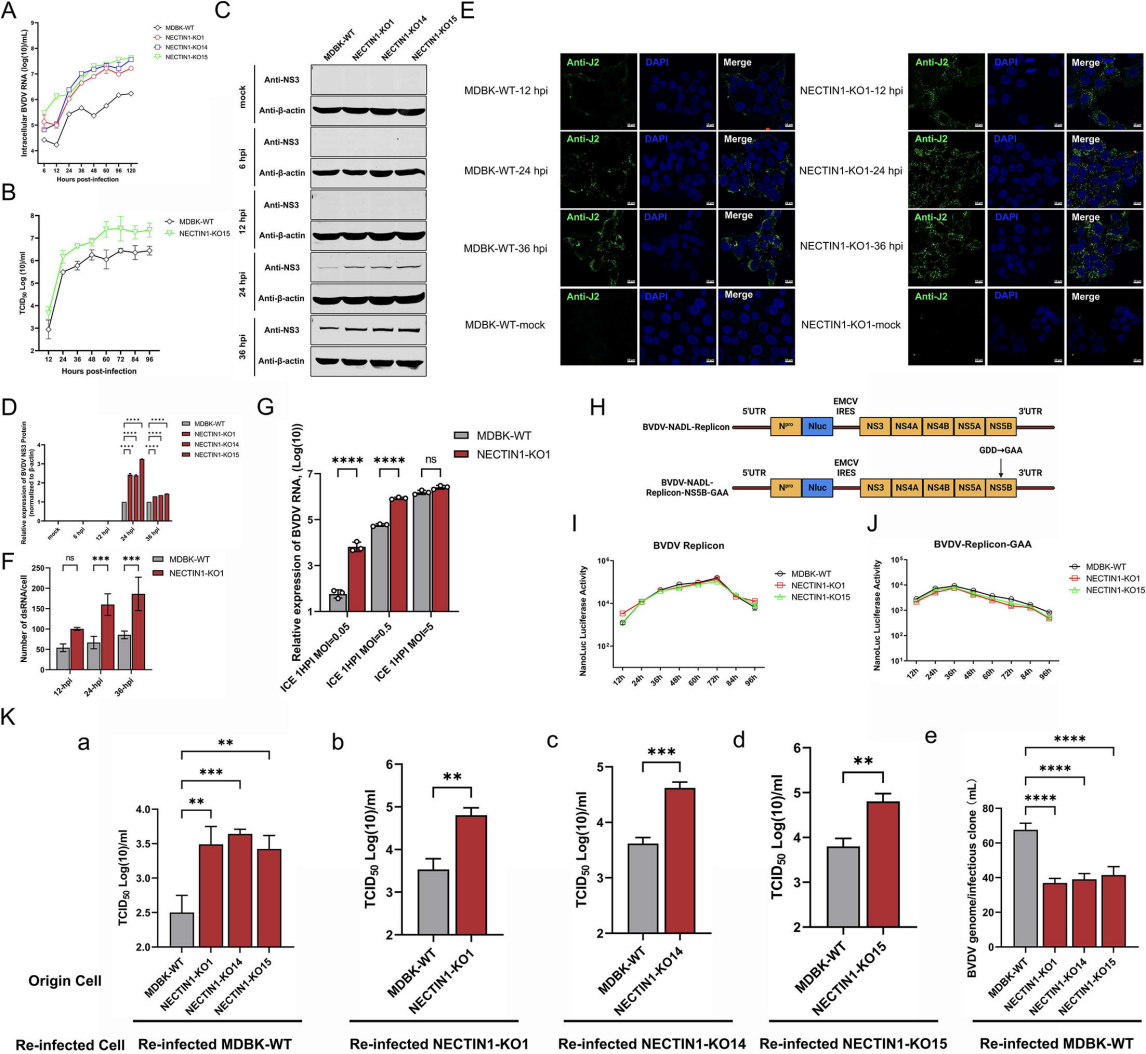
NECTIN1 inhibits attachment during the BVDV life cycle. (**A**) qPCR quantification of intracellular BVDV RNA levels in MDBK-WT and NECTIN1 knockout cell lines at different time points. MDBK-WT cells were used as controls. The cells were infected at an MOI of 1. (**B**) TCID_50_ identification of BVDV titers in MDBK-WT and NECTIN1 knockout cell lines at different time points. MDBK-WT cells were used as controls. The cells were infected at an MOI of 1. (**C**) Western blot detection of BVDV protein levels in MDBK-WT and NECTIN1 knockout cell lines. (**D**) Grayscale analysis of the western blot data. (**E and F**) IFA detection of BVDV dsRNA signal and the number observation of dsRNAs in single cells in the MDBK-WT and NECTIN1 knockout cell lines. Scale bar, 10 µm. (**G**) qPCR quantification of BVDV RNA levels in MDBK-WT and NECTIN1 knockout cell lines at different MOI (0.05, 0.5, and 5) in the binding assay. (**H**) Schematic diagram of the construction of the BVDV-NADL replicon and the BVDV-NADL-NS5B-GAA replicon mutant. (**I**) NanoLuc identification of the replication level of the BVDV-Nanoluc replicon in NECTIN1 knockout cell lines. MDBK-WT cells were used as controls. (**J**) NanoLuc identification of the replication level of the BVDV-Nanoluc-NS5B-GAA replicon in NECTIN1 knockout cell lines. (**K**) qPCR and TCID_50_ determination of the effect of NECTIN1 on the release of infectious virions after BVDV infection. MDBK-WT cells were used as controls. The cells were infected at an MOI of 1.

To elucidate the detailed mechanisms underlying the NECTIN1-mediated inhibition of BVDV infection, we compared the multi-step growth curves of BVDV in MDBK-WT and NECTIN1 knockout cell clones. As early as 6 hpi, the BVDV RNA levels in the NECTIN1 knockout cell clones were approximately 10-fold greater than those in the MDBK-WT cells (Fig. 5A). The TCID_50_ results showed that as early as 12 hpi, the number of infectious virions produced in NECTIN1 KO15 cell was approximately 10 times greater than that in MDBK-WT cells (Fig. 5B). In addition, compared with MDBK-WT cells, BVDV-infected NECTIN1 knockout cell clones produced more viral progeny. Consistently, the BVDV NS3 protein expression level was greater in the NECTIN1 knockout cell clones throughout the entire infection course (Fig. 5C and D). Double-strand RNA (dsRNA) detection revealed that the dsRNA signal in the NECTIN1 knockout cell clones was much stronger than that in the MDBK-WT cells (Fig. 5E and F). These results suggest that NECTIN1 influences BVDV replication at the early stage of infection. To further confirm the above results, we performed a BVDV binding assay and found that NECTIN1 could restrict BVDV binding to the cell surface (Fig. 5G). To determine whether NECTIN1 also inhibits viral replication and translation, we used the Nanoluc luciferase reporter BVDV replicon system to bypass the entry step (Fig. 5H). As described previously (43), the BVDV structural protein capsid, E^rns^, E1, and E2, and nonstructural proteins P7 and NS2 were replaced with the Nanoluc reporter gene (Fig. 5H). As shown in Fig. 5I, the Nanoluc luciferase activity in the MDBK-WT and NECTIN1 knockout cell clones was comparable, indicating that BVDV replication and protein translation were not influenced by NECTIN1. To further demonstrate the functions of this protein in viral translation, we constructed a BVDV replicon mutant (NS5B-GAA) that failed to replicate but retained the translated viral protein using the input viral RNA as a template. We found that there was no significant difference between the two groups and the NanoLuc signal began to decrease at approximately 48 h post-transfection due to a lack of replication (Fig. 5J). Finally, we investigated the impacts of NECTIN1 on BVDV infectious particle production. As shown in Fig. 5K, upon infection with the viral particles produced from each cell clone at an MOI of 1, we observed that the infected NECTIN1 knockout cell clones produced more infectious virus particles, irrespective of the origin of the virions. Surprisingly, by quantifying the specific infectivity defined as the ration of the number of genomes per TCID_50_, it was found that the specific infectivity of virions produced from WT cells was lower than that of virions produced from the NECTIN1 knockout cell clones (Fig. 5K-e). In conclusion, we found that NECTIN1 primarily affected the attachment stage of the BVDV replication cycle, and the virions produced from the NECTIN1-knockout cell clone were more infectious.

### NECTIN1 inhibits BVDV infection by competing with CD46 for binding to BVDV E2

**Fig 6:**
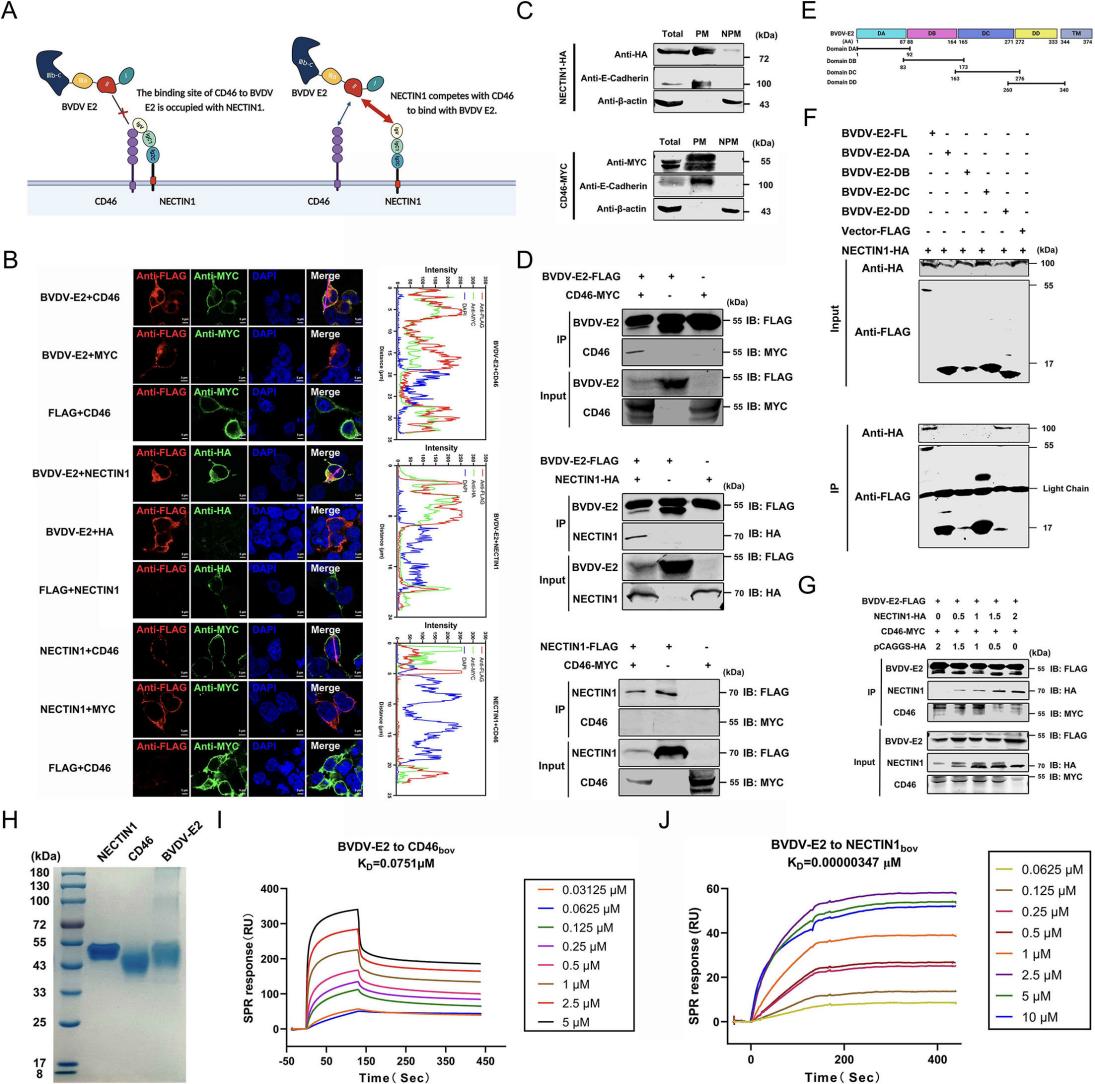
NECTIN1 competes with CD46 for binding to BVDV E2. (**A**) Schematic of the interaction between NECTIN1, BVDV-E2, and CD46. (**B**) Confocal identification of BVDV-E2 and CD46, BVDV-E2 and NECTIN1, and NECTIN1 and CD46. MYC-vector, HA-vector, and FLAG-vector were used as controls. The fluorescence signal in the pink line area was analyzed by the intensity peak diagram. Scale bar, 5 µm. (**C**) Western blot identification of the expression levels of NECTIN1 and CD46 on the plasma membrane. (**D**) Western blot analysis of the interactions between BVDV-E2 and CD46, between BVDV-E2 and NECTIN1, and between CD46 and NECTIN1. MYC-vector, HA-vector, and FLAG-vector were used as controls. (**E**) The schematic diagram of the four domains of BVDV E2. (**F**) Western blot analysis of the interactions between the four domains of BVDV-E2 and NECTIN1. (**G**) Western blot analysis of the interaction between CD46, NECTIN1, and BVDV-E2. HA-vector was used as a control. (**H**) SDS-PAGE analysis of the expression of NECTIN1, CD46, and BVDV E2. (**I**) SPR identification of the binding affinity of BVDV E2 for CD46. (**J**) SPR identification of the binding affinity of BVDV E2 for NECTIN1.

Based on the above results, we demonstrated that NECTIN1 inhibits BVDV infection mainly by interfering with the binding between virions and target cells. Since NECTIN1 and CD46 share a similar Ig-like protein structure, we hypothesized that NECTIN1 and BVDV E2 bind competitively to CD46 or that NECTIN1 and CD46 bind competitively to BVDV E2 (Fig. 6A). Confocal imaging also revealed that E2 colocalized with CD46 and NECTIN1, but the colocalization between NECTIN1 and CD46 was not obvious (Fig. 6B). In addition, as the results shown in Fig. 6C, we detected CD46 and NECTIN1 mainly expressed on plasma membrane by western blot after plasma membrane separation. To this end, we performed a coimmunoprecipitation (Co-IP) assay to determine the interaction between CD46, E2, and NECTIN1. Co-IP revealed that BVDV E2 could interact with both NECTIN1 and CD46, whereas no interaction between NECTIN1 and CD46 was detected (Fig. 6D). The results above demonstrated that BVDV E2 interacted with NECTIN1, and the IgV domain of NECTIN1 was key for restricting BVDV infection. Besides, BVDV E2 was previously divided into four domains namely domain DA, domain DB, domain DC, and domain DD (Fig. 6E) (44). To identify the domains required for BVDV E2 binding with NECTIN1, we first predicted NECTIN1-bovine and BVDV E2 protein model by AlphaFold 3. The docking result shown in Fig. S1, domain DD (272 AA and 333 AA) within BVDV E2 was likely to be the key region mediating the binding with NECTIN1. The three-dimensional schematic diagram showed that NECTIN1 (blue) can stably bind with BVDV E2 (yellow), such as the amino acid Asn77 of NECTIN1 forming hydrogen bonds with the Tyr318 and Arg311 of BVDV E2. To further validate the predicted results, we divided BVDV E2 into four domains according to published data (44) (Fig. 6E). We then transfected them along with NECTIN1 for the Co-IP experiment. The results are shown in Fig. 6F, where NECTIN1 can specifically interact with the domain DD of BVDV E2, which is consistent with the predicted results of AlphaFold 3. From all the above results, we clearly identified the domain of interaction between NECTIN1 and BVDV E2 was domain DD. Furthermore, we examined whether NECTIN1 and CD46 competitively bind to BVDV E2. We co-transfected E2, CD46, and NECTIN1 into cells to determine whether NECTIN1 competitively binds to E2. As shown in Fig. 6G, the amount of CD46 bound to E2 was reduced in the presence of NECTIN1. To further confirm this result, we determined the affinity of NECTIN1 for BVDV E2 or of CD46 for BVDV E2 by SPR. The *K*_*D*_ between NECTIN1 and BVDV E2 was 0.00000347 µM, which was lower than that between CD46 and BVDV E2 indicating that the affinity between NECTIN1 and BVDV E2 is much greater than that between CD46 and BVDV E2 (Fig. 6H through J). All these results indicated that NECTIN1 had a greater affinity than CD46 for E2 and the effect was dose dependence.

### NECTIN1 is a pan-*Flaviviridae* family virus restriction factor for infection

**Fig 7:**
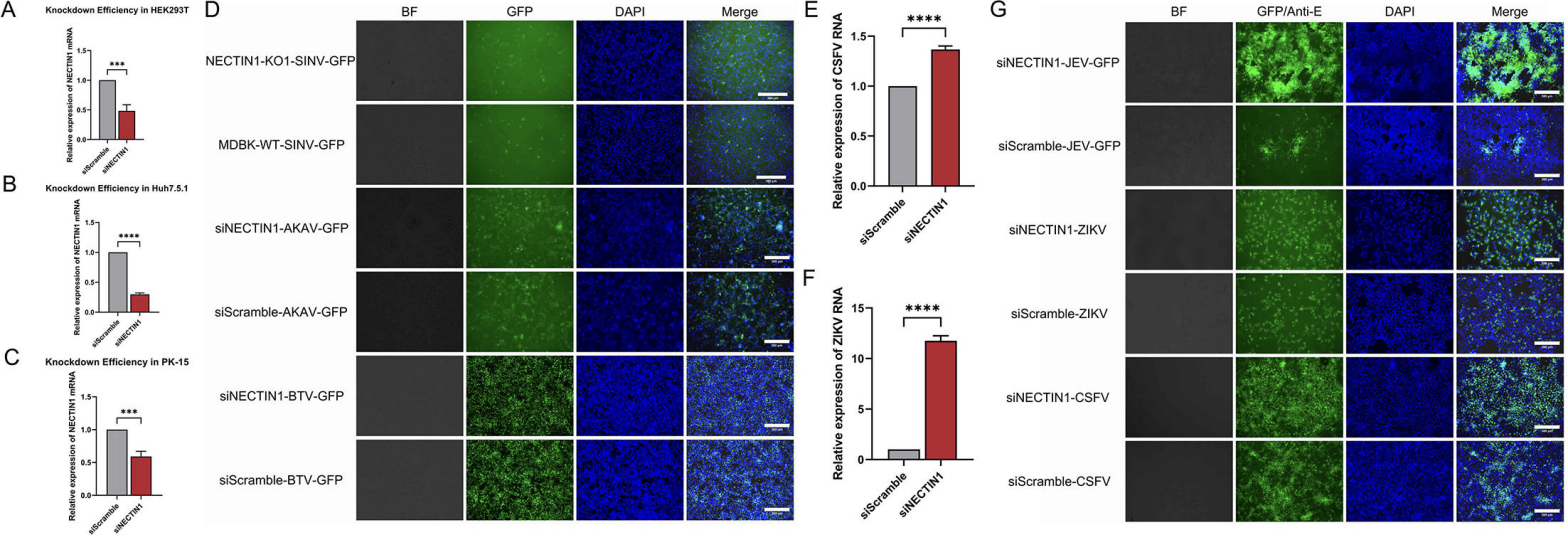
NECTIN1 is a pan-*Flaviviridae* family restriction factor. (**A**) qPCR confirmed the knockdown efficiency of NECTIN1 in HEK293T cells. siScramble was used as a control. (**B**) qPCR revealed the knockdown efficiency of NECTIN1 in Huh7.5.1 cells. siScramble was used as a control. (**C**) qPCR confirmed the knockdown efficiency of NECTIN1 in PK cells. siScramble was used as a control. (**D**) GFP identification of the replication of various viruses following the knockdown of NECTIN1. siScramble was used as a control. Scale bar, 300 µm. (**E**) qPCR analysis of CSFV RNA levels following the knockdown of NECTIN1. siScramble was used as a control. (**F**) qPCR analysis of ZIKV RNA levels following the knockdown of NECTIN1. siScramble was used as a control. (**G**) IFA identification of ZIKV and CSFV replication and GFP identification of JEV replication by following NECTIN1 knockdown. siScramble was used as a control. Scale bar, 300 µm.

These results strongly suggested that NECTIN1 acted as a restriction factor for BVDV infection and competed with the BVDV receptor CD46 for binding to BVDV E2. Given that BVDV belongs to the *Flaviviridae* family and is a single-stranded positive-sense RNA virus, and NECTIN1 was highly expressed in different bovine cell types (Fig. S2A). We hypothesized that NECTIN1 might serve as a pan-restriction factor for the *Flaviviridae* family or even more broadly for RNA viruses. To test this hypothesis, we analyzed the sequences of NECTIN1 from different species and the result showed that NECTIN1 is highly conserved among different species, with a sequence consistency of approximately 93% (Fig. S2B). Then we infected the corresponding host cells with multiple viruses after knocking down NECTIN1. The qPCR analysis that NECTIN1 mRNA levels were significantly reduced in HEK293T, Huh7.5.1, and PK-15 cells following NECTIN1 siRNA transfection (Fig. 7A, B, and C), but GFP signal analysis showed no significant differences in these cells infected with SINV-GFP, AKAV-GFP, or BTV-GFP (Fig. 7D). In addition, both qPCR and IFA revealed that the RNA and protein levels of CSFV and ZIKV were elevated following NECTIN1 knockdown. The replication of JEV-GFP in NECTIN1 knockdown cells was enhanced (Fig. 7E, F, and G). All these findings suggested that NECTIN1 functions as a pan-*Flaviviridae* family restriction factor.

## DISCUSSION

In our study, we identified NECTIN1 as a restriction factor for BVDV despite its structural similarity to CD46. Unlike CD46, NECTIN1 does not act as a receptor for BVDV but rather limits viral replication. Although NECTIN1 has been previously implicated as a receptor for alpha herpesviruses and a receptor-associated protein in poliovirus infection, our findings reveal a novel role for NECTIN1 in the virus life cycle by influencing BVDV infection dynamics.

There was an interesting result that BVDV was concentrated in specific areas and had colocalization with NECTIN1 (Fig. 2K). The previous study reported that NECTIN1 can interact with HSV-1gD to direct the virion internalization (45). According to the previous reports along with our results, we hypothesized that NECTIN1 could interact with BVDV E2 protein to direct it into a certain area for degradation and other processes, thereby limiting the BVDV lifecycle and new investigations are warranted to understand the mechanisms underlying the observation.

Given the structural similarity between NECTIN1 and other members of these families, we investigated whether they exhibit similar functions in BVDV replication, suggesting independent roles for each member. Further investigation into the specific mechanisms underlying their interactions with viruses may provide valuable insights into the complex dynamics of BVDV infection. NECTIN1 belongs to the nectin cell adhesion molecule family and is characterized by its extracellular region comprising three Ig-like domains, a single transmembrane region, and a cytoplasmic tail region (1). The NECTIN cell adhesion molecule family, which includes NECTIN1 to NECTIN4, and the NECTIN-like (NECL) molecular protein family, which consists of NECL1 to NECL5, play crucial roles in cell-cell adhesion through *cis*- and *trans*-interactions (1, 2). Given the structural similarity between NECTIN1 and other members of these families, we sought to investigate whether they exhibit similar functions in BVDV replication. As shown in Fig. 3C and D, despite structural similarities between NECTIN1 and other family members, the knockdown of NECTIN3 and NECTIN4 increased BVDV replication, while the knockdown of NECTIN2 or NECL5 did not influence the BVDV replication. Double knockdown of NECTIN family proteins did not significantly alter BVDV replication compared with the single knockdown, indicating the diverse roles of the NECTIN cell adhesion molecule family in BVDV replication.

Furthermore, we investigated the functional domains of NECTIN1 involved in inhibiting BVDV infection. Our results demonstrated that deletion of the IgV domain (NECTIN1-ΔIgV) had no significant effect on BVDV replication, highlighting the importance of this domain in mediating the interaction with BVDV E2. The transmembrane (TM) domain of NECTIN1 is responsible for its localization in the cell membrane, while the extracellular domain comprises IgV, IgC1, and IgC2. Therefore, we suspected that NECTIN1-ΔTM may fail to localize to the cell membrane and extend its extracellular region outside the cell. A previous study showed that the IgV domain of NECTIN1 is primarily involved in the interaction with HSV-1 gD, further supporting its potential interaction with viral proteins (46). In summary, we determined that IgV is the key domain of NECTIN1 that inhibits BVDV infection. Future research will focus on further characterizing the role of the NECTIN1 IgV domain in interacting with BVDV E2 and elucidating the specific amino acids critical for this interaction.

The above findings suggested that NECTIN1 inhibited BVDV replication and that the IgV of NECTIN1 could interact with BVDV E2. Previous studies have shown that NECTIN family proteins can interact with various Ig-like proteins such as DNAM1/CD226, CRTAM/CD355, Tactile/CD96, and TIGIT (47–50). Our findings indicated that NECTIN1 plays a crucial role in inhibiting BVDV infection during the viral attachment stage. We hypothesized that NECTIN1 could interact with the Ig-like protein CD46, the primary receptor for BVDV, and potentially disrupt the interaction between CD46 and BVDV E2. This hypothesis is shown in Fig. 6A, and the relationship among NECTIN1, CD46, and BVDV E2 was investigated by Co-IP, IFA, and SPR. The results showed that NECTIN1 could compete with CD46 to bind BVDV E2 and that the affinity of BVDV E2 for NECTIN1 was greater than that of BVDV E2 for CD46. According to the SPR results, it was too difficult for the protein to dissociate after BVDV E2 bound to NECTIN1. Furthermore, BVDV E2 domain DD was identified as a key domain of BVDV E2 binding with NECTIN1. However, BVDV E2 domain II was the key domain of BVDV E2 interacting with CD46 (51). Therefore, we hypothesized that after binding to NECTIN1, the conformational structure of BVDV E2 is influenced, resulting in the non-exposure of the CD46 binding site, thereby limiting the binding of CD46 to BVDV E2. These results suggested that NECTIN1 may act as a “sticky trap” during BVDV infection and inhibit BVDV attachment.

During the siRNA pool screening, we also identified several receptors for viruses belonging to the *Flaviviridae* family, such as CD81, CD209, and AXL, all of which play a role in BVDV infection of host cells. CD81, a receptor for HCV, has a similar inhibitory effect on BVDV replication, suggesting a potential pan-receptor role for CD81 in the *Flaviviridae* family (38). Previous studies have shown that AXL and CD209 are ZIKV receptors, and in skin cells, AXL is much more efficient than CD209, TYRO3, and TIM in promoting ZIKV infection (24, 26, 52–54). Notably, NECTIN1, along with the abovementioned proteins, belongs to the Ig-like protein family, and all these results showed that NECTIN1 had highly conserved sequences across different species. The phenomenon led us to hypothesize that NECTIN1 may function as a pan-restriction factor for the *Flaviviridae* family. To investigate this hypothesis, we examined the impact of NECTIN1 on the replication of CSFV, another member of the *Pestivirus* genus, in PK-15 cells, and ZIKV/JEV, a member of the *Flavivirus* genus. Our results demonstrated that NECTIN1 also restricted CSFV, JEV, and ZIKV replication. Furthermore, no significant differences were detected between cells treated with siNECTIN1 and those treated with siScramble after infection with BTV, a double-strand RNA virus, AKAV, a negative-sense single-stranded RNA virus, or SINV, a positive-sense single-stranded RNA virus. Based on these findings, we propose that NECTIN1 acts as a pan-restriction factor for the *Flaviviridae* family. In addition, our findings indicate that NECTIN1 serves as a pan-BVDV restriction factor by impeding BVDV attachment during the viral life cycle.

## SUPPLEMENTAL MATERIAL

Fig. S1 mbio.02708-24-s0001.tiff AlphaFold 3 prediction results of the key structural domains of BVDV E2 protein binding to NECTIN1.

Fig. S2 mbio.02708-24-s0002.tiff The expression levels of NECTIN1 in bovine cells and the sequence similarity between different species.

Supplemental legends mbio.02708-24-s0003.docx Legends for Fig. S1 and S2.

ASM does not own the copyrights to Supplemental Material that may be linked to, or accessed through, an article. The authors have granted ASM a non-exclusive, world-wide license to publish the Supplemental Material files. Please contact the corresponding author directly for reuse.
