# Supplementary figures and images for: Identification of NECTIN1 as a novel restriction factor for flavivirus infection
Source: mBio. 2024 Nov 21;15(12):e02708-24. doi: 10.1128/mbio.02708-24 (PMC11633101; doi:10.1128/mbio.02708-24)

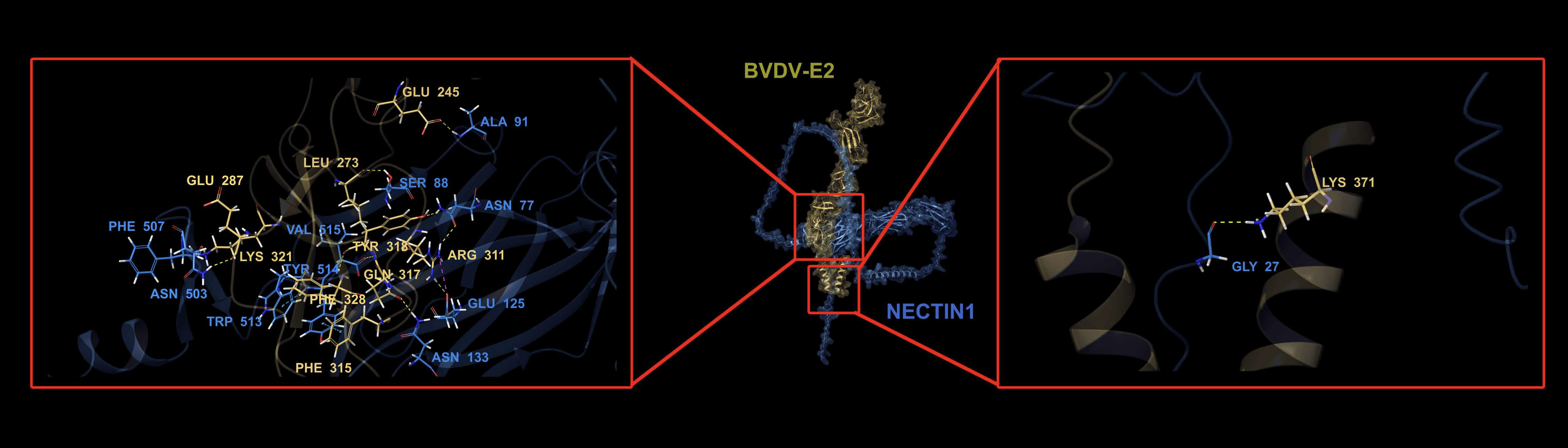

Supplement: Fig. S1 — AlphaFold 3 prediction results of the key structural domains of BVDV E2 protein binding to NECTIN1. [file mbio.02708-24-s0001.tiff]

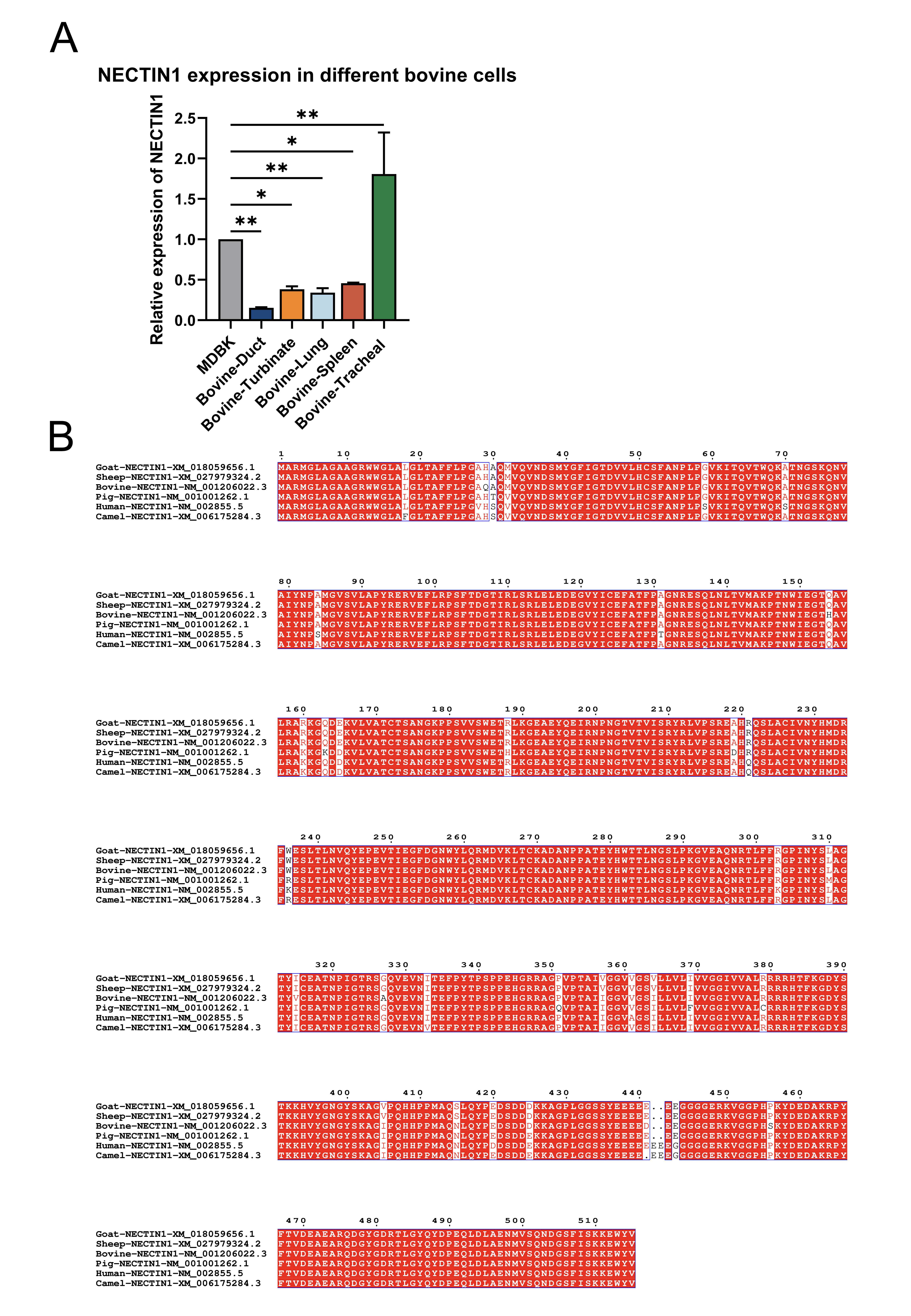

Supplement: Fig. S2 — The expression levels of NECTIN1 in bovine cells and the sequence similarity between different species. [file mbio.02708-24-s0002.tiff]
