# Supplementary material for: Identification of NECTIN1 as a novel restriction factor for flavivirus infection
Source: mBio. 2024 Nov 21;15(12):e02708-24. doi: 10.1128/mbio.02708-24 (PMC11633101; doi:10.1128/mbio.02708-24)
Supplement: Supplemental legends — Legends for Fig. S1 and S2. [file mbio.02708-24-s0003.docx]

**FIG. S1.** **AlphaFold 3 prediction results of the key structural domains of BVDV E2 (yellow) protein binding to NECTIN1 (blue).**

**FIG. S2**. **The expression levels of NECTIN1 in bovine cells and the sequence similarity between different species. (A)**. qPCR analysis for RNA expression level of NECTIN1 in different bovine cells. **(B)**. The schematic diagram of NECTIN1 sequence alignment among different species.
